# Navigating the Quagmire: Comparison and Interpretation of COVID-19 Vaccine Phase 1/2 Clinical Trials

**DOI:** 10.3390/vaccines8040746

**Published:** 2020-12-09

**Authors:** Luca Tudor Giurgea, Matthew James Memoli

**Affiliations:** Laboratory of Infectious Diseases, LID Clinical Studies Unit, Division of Intramural Research, National Institute of Allergy and Infectious Diseases, National Institutes of Health, Bethesda, MD 20892, USA; memolim@niaid.nih.gov

**Keywords:** COVID-19, SARS-COV-2, vaccines, clinical trials

## Abstract

Vaccines against Coronavirus Disease 2019 Originated-19) have been developed with unprecedented rapidity, many utilizing novel strategies. As of November 2020, a series of publications have outlined the results of phase 1/2 studies of nine different vaccines planned to move forward to phase 3 trials. The results are encouraging, demonstrating a paucity of severe or serious adverse events and robust induction of antibody titers. Determination of the vaccine candidates with the highest protective efficacy and best adverse event profiles will be essential in refining public health strategies. However, differences in study design and reporting of data make comparisons of existing phase 1/2 studies difficult. With respect to safety, studies have variable follow-up times and may use different definitions for adverse events. Immunogenicity outcomes are even more inconsistent, with variations in timepoints and critical differences in the types of antibodies studied as well as methodological differences in assays. Furthermore, the correlates of protection in COVID-19 are not known. Harmonization of phase 3 trial designs and use of objective and meaningful clinical outcomes will be crucial in streamlining future global responses to the pandemic.

## 1. Introduction

The COVID-19 pandemic continues to spread across much of the world, despite efforts by individuals and governments to curb transmission of the virus [1]. Medical systems in multiple developed nations have been pushed to their limits, overwhelming healthcare workers and depleting resources [2,3]. Consequently, an international collaborative effort between governments, academic research institutions, and private companies was launched during the early stages of the pandemic to develop vaccines with unprecedented rapidity [4]. By 28 November 2020, clinical trials of nine promising COVID-19 vaccines (chosen for further phase 3 trials or development) have been published in high-profile medical and scientific journals [5,6,7,8,9,10,11,12,13,14]. The purpose of these early trials has been to establish safety, but also to demonstrate some degree of immunogenicity. Large investments have been made to expedite availability of promising vaccine candidates to the public through rapid initiation of phase 3 trials and manufacturing of large amounts of vaccine product prior to determination of vaccine effectiveness. Generous funding of COVID-19 vaccines permitted the use of novel vaccine platforms, many of which made use of rapidly available sequencing information to develop products more quickly than traditional approaches which require growth of virus in biosafety level 3 facilities. While the scientific and regulatory communities are eagerly awaiting the results of phase 3 efficacy trials before approval of vaccines, some have advocated advancing vaccine candidates to market prematurely. In the face of this pressure, a critical comparison of available phase 1/2 study data was performed.

## 2. COVID-19 Vaccine Candidates

Starting on 5 May 2020, the first phase 1 clinical trial evaluating a non-replicating adenovirus type-5 (Ad5) vaccine was published (Table 1) [5]. This was followed by a phase 2 study in July 2020 [7]. Two more trials were published in July 2020 demonstrating safety and immunogenicity of an mRNA vaccine platform (mRNA-1273) and a chimpanzee adenovirus-vectored vaccine (ChAdOx1 nCoV-19) [6,8]. In August 2020, the first trial assessing an inactivated COVID-19 vaccine was published [9]. Following this study, two more were published in September 2020: a recombinant SARS-COV-2 nanoparticle full-length S protein vaccine (NVX-CoV2373) and a combination recombinant adenovirus 26 (rAd26) and rAd5, named Gam-COVID-Vac [10,11]. Data on two additional mRNA vaccines (BNT162b2 and BNT162b1) were published on 13 October with BNT162b2 chosen to progress to stage 3 trials [12], though data on BNT162b1 had been already published earlier [15,16]. Since BNT162b1 is not planned to undergo phase 3 trials, it was not included in the comparison tables in this review. Then, on 15 October, phase 1 and 2 data were published demonstrating safety and immunogenicity of another inactivated virus vaccine (BBIBP-CorV) [13]. Most recently, a phase 1/2 study assessing the efficacy of another inactivated, aluminum hydroxide-adjuvanted vaccine candidate (CoronaVac) was published [14], while other early phase clinical trial manuscripts like Janssen/Johnson & Johnson’s adenovirus 26 based vector vaccine (Ad26.COV2.S) [17], CureVac’s mRNA-based vaccine (CVnCoV) [18], and Medicago’s virus-like particle vaccine are available in preprint [19]. These have not been included in the tables or analysis in this review and are likely to be joined in the coming months by many other vaccine candidates in current early clinical trials.

Forty-eight vaccines are listed by the WHO as being in current clinical evaluation [20]. Each of the vaccine platforms has important caveats and considerations for their use, stemming from the scientific principles underlying the technology, which have been thoroughly reviewed elsewhere [21]. For example, since mRNA has a propensity to degrade rapidly, most mRNA-based vaccines (including the two reviewed here) require frozen storage to maintain stability [6,12,22]. Due to the novelty of the technology, there is limited infrastructure available to support mass vaccination, particularly in resource-poor settings. However, mRNA vaccines can be developed quickly in response to novel pathogens using sequencing data without relying on isolation of the pathogen [23]. Vectored-vaccines can similarly be prepared using sequencing data, but the immunogenicity and likely efficacy of vectored-vaccines based on human endemic adenoviruses may be impacted by pre-existing immunity against the vector itself [7]. ChAdOx1 nCoV-19 bypasses this concern by using a chimpanzee adenovirus vector but may still be limited from use as a booster vaccine by development of immunity against the vector after the first dose [24,25]. For this reason, Gam-COVID-Vac uses a strategy combining two different adenovirus vectors [11,26]. The inactivated vaccines have the potential advantage of inducing immunity against additional targets beyond the S protein but require growth of live virus in biosafety level-3 facilities [27]. Recombinant protein vaccines can also be produced using sequencing data, but often require modification of proteins to support stabilization and proper conformation (particularly of membrane proteins) which may modify the induced immune response [28]. At the time of writing, phase 3 trials evaluating many of these vaccines are well underway as is manufacturing, with others planned to enter phase 3 trials in the near future. Well-designed phase 3 trials involving tens of thousands of participants, control groups, randomization, and blinding will be able to more definitively answer questions of efficacy and safety than phase 1/2 trials.

## 3. COVID-19 Vaccine Phase 1/2 Safety Data

The primary purpose of phase 1 trials is to determine a desirable dose while closely monitoring for common severe adverse events. True differences in side-effect profiles may be attributed to vaccine platforms, vaccine antigen, dosing, scheduling, and use of adjuvants. The vaccines evaluated in these initial phase 1/2 trials used various platforms and dosing schedules, and while some used adjuvants, others did not. Typically, phase 1 trials are small. However, the enrollment in COVID-19 vaccine studies varied from as low as 45 to as high as 1067, in a phase 1/2. This means that the power to detect adverse events (and also immunogenicity) also varied tremendously by study. It is important to note that different vaccination doses/formulations/schedules were tested, so that despite overall large numbers for phase 1 studies, the vaccination strategy chosen for further development may have only been given to as few as 12 participants. Adverse events (AEs) are typically graded by severity on a scale of 1–5. Guidance is given for grading of local and systemic symptoms, vital sign abnormalities, and laboratory abnormalities [29]. The FDA has an additional classification for serious adverse events, defined as those that cause hospitalization or prolonged hospitalization, or those that result in disability, congenital anomaly, life-threatening condition, or death. All of the studies reported an absence of any serious adverse events but most reported mild or moderate (grade 1 or 2) AEs in the majority of participants, though the proportion varied considerably by study. Some vaccines, like BBIBP, were associated with AEs in only a minority of patients while others, like Gam-COVID-Vac, reported mild hyperthermia in 95% of participants. However, in this study, hyperthermia was defined as a temperature greater than 37 °C which is in contrast to the cut-off of 38 °C used in most other studies, outlining the impact of differences in reporting standards across countries in comparisons of outcomes.

The safety results, summarized in Table 2, were not reported in a standardized manner further confounding a rigorous comparison of safety profiles among vaccine candidates. There are dissimilarities in vaccination and timing of follow-up, which varied from two to four weeks post-vaccination. Severe or grade 3 AEs were generally rare. The phase 1 Ad5-vectored COVID-19 vaccine study reported 17% of participants experiencing grade 3 AEs with the high dose vaccine, but this was lowered to 1% during the phase 2 trial using a lower dose. BNT162b2 was associated with grade 3 AEs in 8.3% of participants and ChAdOx1 nCov-19 was also associated with a small amount of grade 3 AEs, though the exact number was not mentioned. The three inactivated virus vaccines appeared to be best tolerated, having the three lowest rates of AEs out of all nine candidates. In summary, all the vaccine candidates to date were reasonably well-tolerated and were not associated with common severe side-effects. However, larger, phase 3 studies conducted over longer periods of time will be necessary to determine if more uncommon side effects may be present.

## 4. COVID-19 Vaccine Phase 1/2 Immunogenicity Data

As opposed to the focus of safety of phase 1 trials, the purpose of phase 2 trials is to get a better sense of a vaccine’s potential efficacy. Some investigators opted to design combination phase 1/2 trials which recruit higher numbers of patients in order to simultaneously and expeditiously address questions of safety and efficacy. Since efficacy, typically measured as prevention of disease or infection, requires large numbers of participants, surrogate endpoints of immunogenicity were used instead. The phase 1 trials also assessed immunogenicity as a secondary endpoint allowing for comparisons between studies, but the difficulties encountered in comparing safety outcomes are only accentuated in analysis of immunogenicity outcomes. It is important to note, while measures of immunogenicity are likely to correlate with protection, they have not been yet demonstrated to do so. There is no scientific consensus on the most meaningful measures of immunogenicity, or the most accurate assays to assess them. In general, measurement of antibodies against Spike protein (S protein), which is essential for SARS-COV-2′s ability to bind and enter host cells, has been performed (Table 3). Some investigators looked at more specific antibodies targeting the receptor binding domain (RBD) of the S protein while others argue that neutralizing antibodies are more important (Table 4) [13]. Some investigators performed assays for antibodies not used in other papers, including targeting antibody against whole SARS-COV-2 antigen and antibody against the S1-subunit, which were not included in Table 3. Comparisons of immunogenicity are difficult across studies assessing different antibodies or even those assessing the same type of antibodies but using different assays. Moreover, the lack of standardization questions any comparisons across studies that use similar assays. For example, anti-RBD antibody titers varied from as low as 571 to as high as 371,271. Neutralization titer variation was not as drastic, but similar differences in assays (pseudovirus neutralization, SARS-CoV-2 neutralization, microneutralization) and techniques (across plaque reduction neutralization testing) were present. Comparisons can be assisted through standardization of post-vaccination titers to baseline titers, though these ratios are highly dependent on the lower bounds of detection of the assay (since antibody levels were generally undetectable at baseline). More accurate comparisons can be achieved through standardization of post-vaccination titers to antibody titers of convalescent COVID-19 patients. This strategy may be confounded by differences in the convalescent patient population, since it has been demonstrated that more severe cases are associated with higher antibody titers [30]. Furthermore, the timing of immunogenicity endpoints varied across studies and some studied immunogenicity outcomes only in a sub-group population. Consequently, it is impractical to quantitatively compare immunogenicity outcomes among COVID-19 vaccine studies. Instead, we can conclude that all vaccines induced antibody production in participants and, in a few studies that checked, post-vaccination titers were higher than titers in convalescent COVID-19 patients. The adenovirus-vectored vaccine studies also differentiated participants who had pre-existing immunity against the vector and found the response to be poorer in those with high antibody titers against adenovirus. Some studies reported the proportion of participants who had a rise in antibody titers (which may be an additional predictor of efficacy compared to median or mean antibody titers across the entire study population). Generally, high proportions of participants responded to all vaccine candidates, with exception of the Ad5-vectored COVID-19 vaccine, which resulted in only 47% of participants developing neutralizing antibodies despite 97% developing anti-RBD antibodies and CoronaVac, which induced neutralizing antibodies in only 25% of participants in phase 1 (but 94.1% in phase 2). Similarly, most vaccines demonstrated induction of T-cell responses (Table 5), which may have more cross-reactive potential than antibodies [31,32,33,34,35], but the type of assay used and the reporting of results varied significantly. These discrepancies are of unclear significance but bring us to a critical and yet unanswered question: what are the correlates of protection in COVID-19? Without understanding the correlates of protection, it is impossible to currently address questions regarding vaccine-associated protection, risk of COVID-19 reinfection, herd immunity, and the possibility of elimination of SARS-COV-2 from the human population.

## 5. Discussion

To better understand correlates of protection in COVID-19, there are three key aspects that need to be addressed: Which antibodies correlate with protection? How much antibody is necessary for protection? What degree of protection can be expected exclusively with specific titers of antibody and in the absence of other forms of immunity? For example, in influenza, a titer of 40 or greater of hemagglutinin inhibiting antibodies correlates with 50% protection against disease and has been used to dictate vaccine design [36,37]. More recently, neuraminidase inhibiting antibodies were shown to be a better predictor of outcomes with influenza [38,39,40]. Large, prospective studies following participants with known antibody titers (and ideally, other immune biomarkers including T-cell immunity) and evaluating infection rates and clinical outcomes are necessary to determine the correlates of protection. The large ongoing and upcoming phase 3 COVID-19 vaccine clinical trials provide excellent opportunities to evaluate correlates of protection, while acknowledging that post-vaccination immunity may differ among vaccines and from post-infection immunity. Furthermore, recognizing the nearly insurmountable aforementioned difficulties in comparing outcomes across phase 1/2 trials, there have been attempts to harmonize some phase 3 trials [41]. Standardization of meaningful, objective clinical outcomes such as mortality, hospitalization, need for mechanical ventilation, as well as other clinical endpoints would help comparisons across studies in the absence of optimal head-to-head trials.

There are additional critical aspects of COVID-19 related immunity that must be considered. A distinction must be made between disease (infection plus symptoms) and infection, which may be asymptomatic but still result in transmission. While reduction of disease severity is a necessary aspect of vaccine-associated protection, a reduction in the rate of asymptomatic infection would be ideal as it would also diminish transmission to non-vaccinated populations, which may be large due to vaccine hesitancy [42,43,44,45]. The majority of vaccines, administered intramuscularly, have focused on induction of serum IgG antibodies which may help to neutralize viremia and attenuate systemic disease. However, mucosal immunity, which relies more heavily on IgA in the upper respiratory tract [46], is not well understood and may be helpful in identifying factors associated with prevention of respiratory virus infections [47]. The duration of post-infection and vaccine-associated protection needs to be further investigated as well. Data to date suggest only mild waning of anti-Spike IgG, anti-RBD IgG, and neutralizing antibody responses in convalescent patients over 3–6 months of follow-up, though serum anti-Spike IgA and anti-RBD IgA decayed much more rapidly [30,48,49,50]. Recent results from the mRNA-1273 phase 1 study also show a similar stability of vaccine-induced antibody responses three months after the second dose [51]. These findings are reassuring, particularly if they can be replicated with vectored vaccines, since the effectiveness of booster doses may be affected by development of anti-vector immunity. Durability data will become particularly valuable once the correlates of protection are better understood. Furthermore, the role of the microbiome in modulating mucosal immunity is underappreciated and poorly understood, particularly in the upper respiratory tract. Recent work has shown the gastrointestinal microbiome regulates constitutive type 1 interferon-mediated antiviral responses [52]. It is also not surprising that COVID-19 is associated with significant changes of the fecal microbiome [53]. Further elucidation of the roles of the respiratory tract microbiome and the mucosal correlates of protection may be important in selecting the most promising and effective vaccine candidates moving forward as well as in the development of future vaccines and therapeutics.

## 6. Conclusions

In the short-term, rapid distribution of all approved vaccine candidates will be necessary while manufacturing capabilities of the best performing vaccines are developed, but selection for universal distribution of the vaccine candidate offering the most durable response, highest efficacy, and the best safety profile will be essential in limiting the spread and impact of the COVID-19 pandemic, an inextricably global problem.

## Figures and Tables

**Table 1:** Characteristics of COVID-19 vaccine candidates and phase 1 or 2 clinical trials.

	Ad5-Vectored COVID-19 Vaccine (Phase 1)	Ad5-Vectored COVID-19 Vaccine (Phase 2)	mRNA-1273	ChAdOx1 nCoV-19	Inactivated COVID-19 Vaccine (Phase 1)	Inactivated COVID-19 Vaccine (Phase 2)	NVX-CoV2373	Gam-COVID-Vac		BNT162b2		BBIBP-CorV (Phase 1)		BBIBP-CorV (Phase 2)	CoronaVac (Phase 1)	CoronaVac (Phase 2)
Vaccine Type	Non-replicating Ad5	Non-replicating Ad5	mRNA	Non-replicating Chimpanzee Ad	Inactivated virus		SARS-CoV-2 nanoparticle trimeric S protein	Frozen rAd26 and rAd5	Lyo rAd26 and rAd5	mRNA		Inactivated virus			Inactivated virus	
Adjuvant	None	None	None	None	Alum		Matrix-M1	None		None		Alum			Alum	
Preferred Dose Regimen	One-dose (1.5 × 10^11^ VP)	One-dose (5 × 10^10^ VP)	Two-dose (100 µg)	One-dose (5 × 10^10^ VP)	Three-dose (5 µg)	Two-dose (5 µg)	Two-dose (5 µg)	Two-dose (10^11^ VP)		Two-dose (30 µg)		Two-dose (4 µg)			Two-dose (3 µg)	
Dose interval			28 days		28 days	14/21 days	21 days	21 days		21 days		28 days		21 days	14 days	
Trial Type	Phase 1	Phase 2	Phase 1	Phase 1/2	Phase 1	Phase 2	Phase 1/2	Phase 1/2		Phase 1		Phase 1		Phase 2	Phase 1	Phase 2
Participants (Total)	108	508	45	1067	96	224	131	76		195		192		448	300	300
Participants (Preferred Dose)	36	129	15	543	24	84/84	29	20	20	12 (ages 18–55)	12 (ages 65–85)	24 (ages 18–59)	24 (ages 60+)	84	120	120
Publication Date	5/22/2020	7/20/2020	7/14/2020	7/20/2020	8/13/20		9/2/2020	9/4/2020		10/14/2020		10/15/2020			11/17/2020	
Affiliation	CanSino Biologics [5]	CanSino Biologics [7]	NIAID and Moderna [6]	University of Oxford and AstraZeneca [8]	WIBP and Sinopharm [9]		Novavax [10]	Gamaleya [11]		BioNTech and Pfizer [12]		BIBP and Sinopharm [13]			Sinovac [14]	

Ad = adenovirus, rAd = recombinant adenovirus, Lyo = lyophilized, VP= viral particles, Alum = aluminum hydroxide, NIAID = National Institute of Allergy and Infectious Diseases, WIBP = Wuhan Institute of Biological Products, Gamaleya = National Centre of Epidemiology and Microbiology, BIBP = Beijing Institute of Biological Products.

**Table 2:** Safety outcomes of COVID-19 vaccine phase 1 or 2 clinical trials.

Ad5-Vectored COVID-19 Vaccine (Phase 1)	Ad5-Vectored COVID-19 Vaccine (Phase 2)	mRNA-1273	ChAdOx1 nCoV-19	Inactivated COVID-19 Vaccine (Phase 1)	Inactivated COVID-19 Vaccine (Phase 2)	NVX-CoV2373	Gam-COVID-Vac	BNT162b2	BBIBP-CorV (Phase 1)	BBIBP-CorV (Phase 2)	CoronaVac (Phase 1)	CoronaVac (Phase 2)
28-day FU		56-day FU (28 after 2nd dose)	28-day FU	84-day FU (28 after 3rd dose)	42–49-day FU (28 after 2nd dose)	35-day FU (14 after 2nd dose)	42-day FU (21 after 2nd dose)	1-month FU after 2nd dose	56-day FU (28 after 2nd dose)	58-day FU (30 after 2nd dose)	42-day FU (28 after 2nd dose)	
Systemic or local AE of any grade (75%). Systemic or local grade 3 AE (17%).	Systemic or local AE of any grade (76%). Systemic or local grade 3 AE (1%).	Systemic grade 1 AE (Dose 1: 53.3%, Dose 2: 20%). Systemic grade 2 AE (Dose 1: 13.3%, Dose 2: 80%). Systemic grade 3 AE (0%). Local grade 1 AE (Dose 1: 73.3%, Dose 2: 66.7%). Local grade2 AE (Dose 1: 13.3%, Dose 2: 26.7%). Local grade 3 AE (0%).	Fatigue (70%), headache (68%), muscle aches (60%), malaise (61%), chills (56%), feeling feverish (51%), documented fever (18%). A small proportion of AE of all types were severe, though AE profile was improved with paracetamol administration.	Systemic grade 1/2 AE (12.5%). Systemic grade 3 AE (0%). Local grade 1/2 AE (4.2%). Local grade 3 AE (0%).	14-day interval: systemic grade 1/2 AE (4.8%). 21-day interval: systemic grade 1/2 AE (4.8%). Systemic grade 3 AE (0%). 14-day interval: local grade 1/2 AE (2.4%). 21-day interval: local grade 1/2 AE (15.5%). Local grade 3 AE (0%).	Systemic grade 1 AE (Dose 1: 40%, Dose 2: 40%). Systemic grade 2 AE (Dose 1: 5%, Dose 2: 20%). Systemic grade 3 AE (Dose 1: 0%, Dose 2: 10%). Local grade 1 AE (Dose 1: 60%, Dose 2: 55%). Local grade 2 AE (Dose 1: 10%, Dose 2: 35%). Local grade 3/4 AE (0%). (approx.)	Grade 1 hyperthermia (95%), headache (45%), asthenia (55%), myalgia/arthralgia (20%), diarrhea (15%), rhinorrhea (20%), loss of appetite (5%), pharyngalgia (5%), malaise (10%), sore throat (10%), nasal congestion (5%), cough (5%), sneezing (5%), pain (40%), hyperthermia (20%), swelling (5%). Grade 2 Hyperthermia (5%), headache (10%), myalgia/arthralgia (5%). Systemic grade 3 AE (0%). Local grade 2/3 AE (0%).	Ages 18–55: systemic or local AE of any grade (41.7%). Systemic or local grade 3 AE. (8.3%) Ages 65–85: systemic or local AE of any grade (25%). Systemic or local grade 3 AE (8.3%).	Ages 18–59: systemic or local grade 1 AE (33%). Systemic or local grade 2 AE (13%). Ages 60+: Systemic or local grade 1 AE (29%).	Systemic or local grade 1 AE (15%). Systemic or local grade 2 AE (2%)	Systemic grade 1 AE (12.5%). Systemic grade 2/3 AE (0%). Local grade 1 AE (16.7%). Local grade 2/3 AE (0%).	Systemic mostly grade 1 AE (15.8%) Local mostly grade 1 AE (23.3%)

FU = follow-up, AE = adverse events, (approx.) = Approximated visually from bar graphs.

**Table 3:** Immunogenicity of vaccines as measured by antibodies against spike and the receptor binding domain.

	Ad5-Vectored COVID-19 Vaccine		mRNA-1273	ChAdOx1 nCoV-19	Inactivated COVID-19 Vaccine (Phase 1/2)	NVX-CoV2373	Gam-COVID-Vac		BNT162b2	BBIBP-CorV (Phase 1/2)	CoronaVac	
Subgroup	Phase 1	Phase 2					Frozen	Lyo			Phase 1	Phase 2
Vaccine Schedule	Day 0	Day 0	Day 0/28	Day 0	Day 0/28/56, 0/14, 0/21	Day 0/21	Day 0/21	Day 0/21	Day 0/21	Day 0/28,0/21	Day 0/14	Day 0/14
Anti-S Ab Assay	ELISA	NP	ELISA	ELISA	NP	ELISA	NP	NP	NP	NP	NP	NP
Anti-S Ab Titer	596.4		782,719	157.1		63,160.0						
Endpoint/Baseline Titer Ratio	*		5975.0	157.1		556.0						
Endpoint/Convalescent Control Titer Ratio	NP		5.5	*		7.6						
% Seroconverted	83%		100%	*		100%						
Timepoint	Day 28		Day 57	Day 28		Day 35						
Anti-RBD Ab Assay	ELISA	ELISA	ELISA	MIA	NP	NP	ELISA	ELISA	MIA	NP	ELISA	ELISA
Anti-RBD Ab Titer	1445.8	571.0	371,271	3182.5			14,703.0	11,143.0	*		465.8	1053.7
Endpoint/Baseline Titer Ratio	*	26.1	2236.6	172			14,703.0	11,143.0	*		5.8	12.9
Endpoint/Convalescent Control Titer Ratio	NP	*	9.8	*			11.6	8.8	*		NP	NP
% Seroconverted	100%	97%	*	*			100%	100%	*		87.5%	97.4%
Timepoint	Day 28	Day 28	Day 57	Day 28			Day 42	Day 42	Day 28		Day 42	Day 42

Lyo = lyophilized, Anti-S = anti-spike, Ab = antibody, ELISA = enzyme-linked immunosorbent assay, MIA = multiplexed immunoassay, * = data not provided, NP = not performed. Data is based upon dosing strategy identified to proceed to next phase of clinical trials, outlined in Table 1.

**Table 4:** Immunogenicity of vaccines as measured by pseudovirus neutralization, SARS-COV-2 neutralization, and microneutralization titers.

	Ad5-Vectored COVID-19 Vaccine		mRNA-1273	ChAdOx1 nCoV-19	Inactivated COVID-19 Vaccine (Phase 1)	Inactivated COVID-19 Vaccine (Phase 2)		NVX-CoV2373	Gam-COVID-Vac		BNT162b2		BBIBP-CorV (Phase 1)		BBIBP-CorV (Phase 2)	CoronaVac	
Subgroup	Phase 1	Phase 2							Frozen	Lyo	Ages 18–55	Ages 65–85	Ages 18–59	Ages 60+		Phase 1	Phase 2
Vaccine Schedule	Day 0	Day 0	Day 0/28	Day 0	Day 0/28/56	Day 0/14	Day 0/21	Day 0/21	Day 0/21	Day 0/21	Day 0/21	Day 0/21	Day 0/28	Day 0/28	Day 0/21	Day 0/14	Day 0/14
Pseudovirus Neutralization Titer	45.6	55.3	231.8	87.9	NP	NP	NP	NP	NP	NP	NP	NP	NP	NP	NP	22.4	84.9
Endpoint/Baseline Titer Ratio	*	10.1	23.2	2.2												*	*
Endpoint/Convalescent Control Titer Ratio	NP	*	2.1	NP												*	*
% Seroconverted	69%	83%	100%	*												41.7%	79.7%
Timepoint	Day 28	Day 28	Day 57	Day 28												Day 28	Day 28
SARS-CoV-2 Neutralization Assay Type	*	*	PRNT_80_	PRNT_50_	PRNT_50_	PRNT_50_	PRNT_50_	NP	NP	NP	fPRNT_50_	fPRNT_50_	*	*	*	Micro CPE Assay	Micro CPE Assay
Neutralization Titer	34.0	18.3	654.3	218.0	206	121	247				361.0	149.0	29.3	18.9	282.7	5.4	23.8
Endpoint/Baseline Titer Ratio	*	4.6	163.6	9.5	41.1	24.1	49.3				36.1	14.9	14.7	9.5	141.4	2.7	11.9
Endpoint/Convalescent Control Titer Ratio	NP	*	4.1	NP	NP	NP	NP				3.8	1.6	NP	NP	NP	NP	NP
% Seroconverted	75%	47%	100%	100%	95.8%	97.6%	97.6%				*	*	100%	92%	100%	25%	94.1%
Timepoint	Day 28	Day 28	Day 43	Day 28	Day 70	Day 28	Day 35				Day 28	Day 28	Day 28	Day 28	Day 49	Day 42	Day 42
Microneutralization Titer	NP	NP	NP	NP	NP	NP	NP	3906.3	49.3	46.0	NP	NP	NP	NP	NP	NP	NP
Endpoint/Baseline Titer Ratio								195.3	39.4	36.8							
Endpoint/Convalescent Control Titer Ratio								4.0	1.5	1.4							
% Seroconverted								100%	100%	100%							
Timepoint								Day 35	Day 42	Day 42							

Lyo = lyophilized, NP = not performed, * = data not provided, PRNT = plaque reduction neutralization test. fPRNT = fluorescent plaque reduction neutralization test. CPE = cytopathic effect. Data is based upon dosing strategy identified to proceed to next phase of clinical trials, outlined in Table 1.

**Table 5:** Immunogenicity of vaccines as measured by T-cell responses.

	Ad5-Vectored COVID-19 Vaccine (Phase 1)	Ad5-Vectored COVID-19 Vaccine (Phase 2)	mRNA-1273	ChAdOx1 nCoV-19	Inactivated COVID-19 Vaccine (Phase 1/2)	NVX-CoV2373	Gam-COVID-Vac		BNT162b2	BBIBP-CorV (Phase 1/2)	CoronaVac (Phase 1)	CoronaVac (Phase 2)
Subgroup							Frozen	Lyo				
T-Cell Response Assay	INF-gamma ELIspot	INF-gamma ELIspot	ICS	INF-gamma ELIspot	NP	ICS	INF-gamma ELISA	INF-gamma ELISA	NP	NP	INF-gamma ELIspot	NP
Proportion with T-Cell Response	83–97%	88%	*	*		*	90%	85%			45.8%	

Lyo = lyophilized, INF= interferon, ELIspot = enzyme-linked immune absorbent spot, NP = not performed, ICS = intracellular cytokine staining. * = data not provided. Data is based upon dosing strategy identified to proceed to next phase of clinical trials, outlined in Table 1.
